# Adult T-cell Leukemia/Lymphoma in Japan: Real-World Treatment Patterns, Healthcare Resource Utilization, and Costs

**DOI:** 10.36469/001c.162558

**Published:** 2026-08-03

**Authors:** Charles Dharmani, Zahid Islam, Pingping Qu, Zoe Jiang, Jingjian Wang, Li Li, Yijing Tao, Lin Song

**Affiliations:** 1 Daiichi Sankyo Inc., Basking Ridge, New Jersey, USA; 2 DeltaMed Solutions, Inc., Somerset, New Jersey, USA; 3 Daiichi Sankyo Co. Ltd., Tokyo, Japan

**Keywords:** adult T-cell leukemia/lymphoma, Medical Data Vision, systemic therapy, line of therapy, patient characteristics, treatment patterns, healthcare resource utilization, treatment costs

## Abstract

**Background:**

Adult T-cell leukemia/lymphoma (ATL) is a rare and aggressive type of peripheral T-cell malignancy caused by the human T-lymphotropic virus type 1 (HTLV-1). Approximately 5 to 10 million people are infected with HTLV-1 worldwide, with high prevalence in Japan. This observational study analyzed patient characteristics, treatment patterns, healthcare resource utilization (HRU), and costs of ATL in Japan.

**Methods:**

A retrospective analysis of ATL patients from the Medical Data Vision database, Sept. 1, 2012–Aug. 31, 2022, was conducted.

**Results:**

Among 1572 ATL patients, 384 received systemic therapy. The mean age at diagnosis was 66.4 years, with 63.7% being ≥65 years; 55.2% patients were females. Excluding cancer, 61% of patients had a Charlson Comorbidity Index score of 0. The most frequently observed comorbidities were congestive heart failure (15.8%), diabetes (14.8%), and peptic ulcer disease (14.6%). Among the treated patients, 37.5% received 1 line of therapy, 26% received 2 lines of therapy, and 36.5% received ≥3 lines. Mogamulizumab was most frequently utilized (60.4%), as monotherapy or in combination regimens. The most common combination regimen in all lines of therapy were cyclophosphamide, doxorubicin, vincristine, and prednisone (CHOP), utilized in 46.6% of patients. Additionally, use of combinations such as VCAP-AMP-VECP, which include vincristine, cyclophosphamide, doxorubicin, and prednisone; adriamycin, manimustine, and prednisone; and vindesine, etoposide, carboplatin, and prednisone, was observed in 33.3% of patients. Initial treatment was discontinued in 74.7% patients; only 3.4% received stem cell transplants. Most (96%) patients were hospitalized, 29.7% had emergency room visits, and 79.4% averaged 1.8 outpatient visits monthly. The mean all-cause healthcare cost per patient per month was $29 538 (¥4 647 300), mainly due to outpatient visits and hospitalizations.

**Conclusion:**

Although real-world studies based on MDV database have certain limitations, the findings of this study provide deeper understanding of the real-world patient characteristics, treatment patterns, HRU, and costs of ATL patients in Japan. The results indicate that patients often encounter high treatment costs and frequent hospitalizations, demonstrating the necessity for advanced regimens and highlighting the unmet need for more effective therapies in ATL patients that may reduce HRU and economic burden in this patient population.

## BACKGROUND

Adult T-cell leukemia/lymphoma (ATL) is a rare and aggressive type of peripheral T-cell malignancy caused by the human T-lymphotropic virus type 1 (HTLV-1).^1,2^ Approximately 5 to 10 million people are infected with HTLV-1 worldwide, with higher prevalence rates in certain geographic regions, including Japan.^3,4^ As of 2020-2021, it was estimated that there were approximately 650 000 HTLV-1 carriers in Japan, with an incidence rate of 3.8 cases per 100 000 person-years reported in 2016.^5,6^ Chronic HTLV-1 infection can lead to ATL, which may develop decades after the initial infection, often with a latency period exceeding 40 to 50 years.^7^ The lifetime risk of developing ATL among HTLV-1 carriers is estimated to range from 2% to 6%.^4,6,8,9^ Several risk factors have been identified, including a high proviral load, male sex, older age, and immunosuppression.^6,8^ Globally, around 2000 to 3000 new cases of ATL are diagnosed each year, with nearly 1000 of those cases occurring in Japan.^10,11^ Most cases in Japan are concentrated in southwestern regions, particularly Kyushu and Okinawa, where HTLV-1 is endemic. The demographics of ATL patients vary significantly by region. In Japan, ATL primarily affects older adults, particularly males from ethnic groups where the HTLV-1 is endemic.^12,13^

ATL is broadly classified into 4 clinical subtypes: acute, lymphoma, chronic, and smoldering.^6^ Acute, lymphoma, and certain unfavorable chronic variants are typically aggressive, whereas smoldering and some chronic variants tend to be indolent.^14^ The prevalence of these clinical subtypes varies considerably across geographic regions.^15,16^ All 4 ATL subtypes are represented in Japan, with approximately 51% to 57% of cases being acute, 19% to 25% classified as lymphoma, 12% to 19% as chronic, and 6% to 11% identified as smoldering.^14^ Indolent ATL is rarely diagnosed outside Japan. A study involving a Caribbean cohort treated in New York revealed that only 3.7% of cases were indolent, while about 90% were classified as acute or lymphomatous ATL.^17,18^

The natural history, clinical presentation, and treatment strategies for ATL vary significantly by disease subtype and geographic region.^19^ In Japan, patients with the more favorable chronic or smoldering forms of ATL are often monitored without chemotherapy, except for topical treatments for skin lesions, until signs of progression to the more aggressive types of ATL appear. For aggressive forms of the disease, treatment options usually include antiviral therapy with zidovudine and interferon-alpha (AZT/IFN), various combinations of chemotherapy, or chemotherapy followed by antiviral therapy. Unfortunately, ATL patients typically have poor prognosis and often develop resistance to first-line chemotherapy treatments. In some aggressive cases, allogeneic hematopoietic stem cell transplantation (allo-HSCT) may offer a potential cure for eligible patients. Additionally, new treatment options, such as anti-CCR4 antibodies, show promise for improving clinical outcomes, particularly in elderly patients.^15,16,20^

Due to the rarity of ATL and its poor prognosis, there is limited understanding of the disease, particularly regarding the characteristics of the patient population and their real-world treatment patterns.^21^ Previous retrospective studies conducted on Japanese patients have evaluated the safety and efficacy of various treatment regimens, as well as the identification of biomarkers and prognostic factors among ATL patients, using registry- and hospital-based datasets.^22^ However, these studies have not utilized large real-world databases, and evidence on the characteristics of ATL patients, treatment patterns by line of therapy, healthcare resource utilization, and costs of treatment is scarce. The Medical Data Vision (MDV) database provides a comprehensive collection of healthcare claims data and Diagnosis Procedure Combination data from hospitals across Japan.^23^ The primary objective of this study was to evaluate the baseline characteristics of ATL patients in Japan and to analyze their treatment patterns, healthcare resource utilization (HRU), and costs using the MDV database.

## METHODS

### Study Design

This real-world observational study retrospectively analyzed a large cohort of ATL patients in Japan, utilizing the MDV database. The cohort identification period included data from September 1, 2012, to August 31, 2022, with a 6-month baseline period.

### Data Source

The MDV database is a large commercial database containing open claims data for approximately 52.37 million patients in Japan, with a significant proportion over 65 years old. It includes data from about 30% of advanced treatment hospitals, collecting over 1 million health claims monthly since April 2008.^24^ MDV provides information on hospitalized and outpatient patients, including *International Classification of Diseases, Tenth Revision* (ICD-10) codes, main disease flags, healthcare costs, and drug details like prescription dates, European Pharmaceutical Marketing Research Association Anatomical Classification of Pharmaceutical Products classification codes, and daily doses. Clinical laboratory test values are also included, featuring test dates, *Japan Laboratory Code Tenth Revision* codes, result values, and units. Death information is limited to in-hospital occurrences only.^24^

### Patient Population and Index Dates

Adult patients (≥18 years) with at least 1 confirmed claim of the earliest diagnosis code of ATL (Disease Codes: 8847374, 8847375, 8847376, 8847377, 8847378, 8847282, 8835876, 8835877, 8842126 corresponding to ICD-10 codes: C915, C795) between September 1, 2012, and August 31, 2022, were identified. The date of the first claim of ATL diagnosis was the index diagnosis date, and a 6-month look-back period prior to the index diagnosis date was determined. Patients were required to be without a non-ATL primary cancer diagnosis ±30 days of the index diagnosis date, and to have at least 1 claim during the 6-month look-back period. Patients were excluded if there was any claim of systemic ATL treatment prior to the index diagnosis date. In addition, patients were required to have ≥1 month of continuous follow-up after the index diagnosis date.

### Continuous Enrollment and Study Period

Continuous enrollment or follow-up in this study was defined as having 1 or more claims every 3 consecutive months. The study period for each patient was from 6 months before ATL diagnosis to death, or the end of continuous enrollment or follow-up. Out-of-hospital deaths and treatment at other institutions were not captured during an individual patient’s follow-up and the patient was censored at the end of last claim.

Patients who have records of receiving ATL-related systemic treatments on or after the ATL diagnosis date were included in the treatment pattern and HRU and cost analysis. The date of the first ATL treatment claim was the index treatment date, and patients were required to have at least 1 claim during the 6-month period prior to the index treatment date and to have at least 1 month of continuous follow-up after the index treatment date. Patients were categorized into 3 mutually exclusive subcohorts based on the total number of lines of therapy observed during follow-up: 1LOT, 2LOT, or ≥3LOT. Line of therapy end date was defined as the earliest of the following events: discontinuation (eg, gap in treatment of ≥60 days), line advance (switch from index treatment to another ATL treatment within ≥30 days after line of therapy index treatment initiation) or augmentation (additional therapies initiated after the initial 30-day window within the line of therapy), end of continuous enrollment or end of study period.

### Line of Therapy

During the follow-up period, ATL-related treatment regimens for the overall treated cohort and LOT subcohorts were identified. A line of therapy regimen was defined as a segment of ATL-related treatment and could be a monotherapy or combination therapy regimen. Among combination regimens, the CHOP-like regimen included combinations with cyclophosphamide, doxorubicin and vincristine while allowing for different corticosteroids (eg, prednisone, methylprednisolone, dexamethasone) within the regimen. Similarly, VCAP-AMP-VECP-like regimen included combinations containing vincristine, cyclophosphamide, doxorubicin, ranimustine, vindesine, etoposide, and carboplatin along with any corticosteroid.

### Statistical Analysis

All statistics in this study are descriptive in nature and provided for the overall treated cohort and the LOT subcohorts, wherever applicable. Continuous variables were summarized with sample size (n), mean, standard deviation (SD), median, 25% percentile (Q1), 75% percentile (Q3), minimum, and maximum. Categorical variables were summarized with frequency and percentage. Missing values were not imputed. Time on treatment was defined as the time from initiation of a line of therapy to end of that line of therapy, with no censoring, with line of therapy end date defined for every patient as described in the Line of Therapy section. Median time on treatment, probability of being on treatment at specific time points, and associated 95% confidence intervals (CIs) were estimated using the Kaplan-Meier method stratified by the top treatment regimen observed at first, second, and third or higher line of therapy. All-cause and ATL-related HRU and associated costs were assessed in the treated cohort and LOT subcohorts. The number of each HRU was calculated as per patient per month (PPPM). Cost was also calculated as PPPM; first in Japanese yen and then converted to US dollars by using the currency conversion rate on January 1, 2025 (US $1 = ¥157.33). All analyses were performed using SAS version 9.4 (SAS Institute Inc.).

## RESULTS

### Patient Characteristics

**Figure 1.**
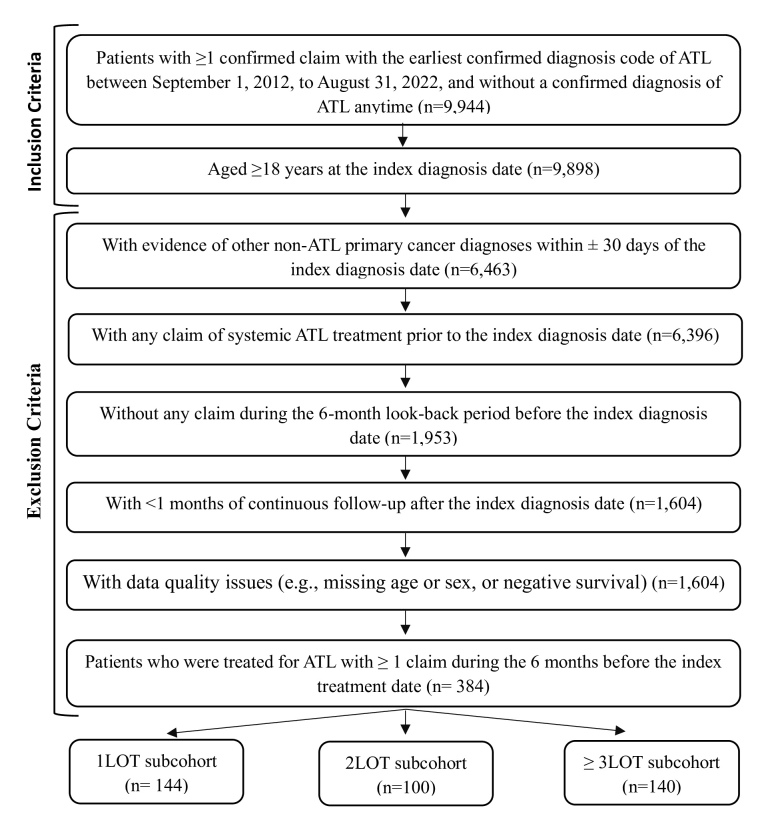
Patient Selection Criteria and Cohort Attrition Abbreviations: ATL, adult T-cell leukemia/lymphoma; LOT, line of therapy; MDV, Medical Data Vision database. Stepwise application of inclusion and exclusion criteria to select the study population (overall cohort) and treated cohort by line of therapy in MDV database, 1 September 2012 to 31 August 2022.

Among 1572 patients meeting the selection criteria in the overall ATL cohort, a total of 1188 patients did not have any record of receiving any systemic therapy. Only 384 patients, who had treatment records of receiving systemic therapy, were selected for further analysis and considered as the treated cohort. Among the treated cohort, 144 received first-line therapy only, while 100 and 140 patients received second-line and third-line therapies, respectively (**Figure 1**). The demographic and baseline clinical characteristics of these patients are presented in **Table 1**.

**Table 1.** Baseline Demographic and Clinical Characteristics of the Study Cohorts and Line of Therapy Subcohorts

**Characteristics**	**Overall (N = 1572)**	**Untreated Overall (N = 1188)**	**Treated**			
**Overall (N = 384)**	**1LOT (N = 144)**	**2LOT (N = 100)**	**≥3LOT (N = 140)**			
Age (years)						
Mean (SD)	66.4 (15.6)	65.4 (16.7)	69.6 (10.9)	72.3 (10.5)	68.5 (12.0)	67.5 (9.9)
Median [Q1, Q3]	69 [59.0, 77.0]	68 [56.0, 78.0]	70 [64.0, 77.0]	74 [67.0, 80.5]	70 [62.5, 77.0]	68 [62.5, 74.0]
Age group (years), n (%)						
18-24	20 (1.3)	19 (1.6)	1 (0.3)	0 (0.0)	1 (1.0)	0 (0.0)
25-39	111 (7.1)	108 (9.1)	3 (0.8)	0 (0.0)	1 (1.0)	2 (1.4)
40-54	181 (11.5)	152 (12.8)	29 (7.6)	9 (6.3)	9 (9.0)	11 (7.9)
55-64	258 (16.4)	192 (16.2)	66 (17.2)	20 (13.9)	19 (19.0)	27 (19.3)
≥65	1002 (63.7)	717 (60.4)	285 (74.2)	115 (79.9)	70 (70.0)	100 (71.4)
Age at index treatment (years)						
n	–	–	384	144	100	140
Mean (SD)	–	–	69.8 (10.9)	72.8 (10.5)	68.6 (12.0)	67.7 (10.0)
Median [Q1, Q3]	–	–	71 [64.0, 77.0]	74[ 67.5, 81.0]	70 [62.5, 77.5]	68 [63.0, 75.0]
Sex, n (%)						
Male	704 (44.8)	521 (43.9)	183 (47.7)	77 (53.5)	53 (53.0)	53 (37.9)
Female	868 (55.2)	667 (56.1)	201 (52.3)	67 (46.5)	47 (47.0)	87 (62.1)
ATL subtypes, n (%)						
Unknown	1215 (77.3)	964 (81.1)	251 (65.4)	97 (67.4)	62 (62.0)	92 (65.7)
Smoldering	123 (7.8)	106 (8.9)	17 (4.4)	9 (6.3)	2 (2.0)	6 (4.3)
Acute	78 (5.0)	26 (2.2)	52 (13.5)	16 (11.1)	14 (14.0)	22 (15.7)
Lymphoma	70 (4.5)	36 (3.0)	34 (8.9)	9 (6.3)	14 (14.0)	11 (7.9)
Chronic	49 (3.1)	41 (3.5)	8 (2.1)	5 (3.5)	1 (1.0)	2 (1.4)
≥2 ATL subtypes	37 (2.4)	15 (1.3)	22 (5.7)	8 (5.6)	7 (7.0)	7 (5.0)
Charlson Comorbidity Index						
Mean (SD)	0.9 (1.5)	0.9 (1.5)	0.9 (1.3)	1.1 (1.5)	0.9 (1.3)	0.8 (1.2)
Median [Q1, Q3]	0 [0.0, 2.0]	0 [0.0, 2.0]	0 [0.0, 2.0]	0 [0.0, 2.0]	0 [0.0, 2.0]	0 [0.0, 2.0]

Abbreviations: ATL, adult T-cell leukemia/lymphoma; LOT, line of therapy; Q, quartile; SD, standard deviation.

For the overall ATL cohort, the mean (SD) age at diagnosis was 66.4 (15.6) years. ATL was more prevalent in older individuals, with 63.7% (1002 patients) being at least 65 years of age. The cohort also had a higher representation of females (55.2%) than males (44.8%). Most patients in the overall cohort had a Charlson Comorbidity Index (CCI) score (excluding cancer) of 0, accounting for 61.1% of the cohort. The most common comorbidity in the overall cohort was congestive heart failure (15.8%), followed by peptic ulcer disease (14.6%), mild liver disease (13.0%), and diabetes without chronic complications (10.0%). Chronic pulmonary disease was observed in 2.4% patients in the overall cohort in 3.6% among those treated with ATL regimens.

The untreated patients had a mean (SD) age of 65.4 (16.7) years. Females were predominant in this group (56.1%) **(Table 1)**. The mean (SD) CCI score was 0.9 (1.5) in this group. Most patients (62.2%) in this group had a CCI score of 0. Congestive heart failure (15.3%), diabetes (14.8%), peptic ulcer disease (12.7%), and mild liver disease (12.2%) were the most frequently observed comorbidities.

Subtype classification was unavailable for 77.3% of all ATL patients. Among cases with identified subtypes, smoldering was the most prevalent (7.8%), followed by acute (5.0%), lymphomatoid (4.5%), and chronic subtypes (3.1%), which represented the lowest proportion **(Table 1)**. For the treated cohort, the younger patients received higher lines of therapy (mean age: **≥**3LOT = 67.5 years vs 1LOT = 72.3 years). Men were predominant in 1LOT (53.5%) and 2LOT (53.0%), while women were predominant in the **≥**3LOT (62.1%) **(Table 1)**. The mean CCI score was lowest in the ≥3LOT group (0.8 [SD:1.2]); 63.6% of the ≥3LOT had a CCI score of 0, while 1LOT and 2LOT had a CCI score of 0 in 52.8% and 57.0% patients, respectively. Like the overall cohort, congestive heart failure, peptic ulcer disease, mild liver disease, and diabetes without chronic complications were the most prevalent comorbidities in all 3 subcohorts.

### Drug Utilization and Treatment Patterns

Of the overall cohort, 384 ATL patients had records of receiving chemotherapy treatment. Among these patients, 37.5% were treated with 1 line of therapy, 26.0% with 2 lines of therapy, and 36.5% received 3 or more lines of therapy during follow-up. The median duration of continuous follow-up for the overall cohort was 8.2 months. The longest duration of continuous follow-up was observed in ≥3LOT subcohort (14.9 months) followed by 2LOT (6.6 months) and 1LOT (3.7 months) (**Table 2**). Combination therapies were more prevalent than monotherapy regimens for ATL across all three lines of therapy. For the first-line combination therapies, the most frequently observed regimens included CHOP and CHOP-like treatments (33.6%), VCAP-AMP-VECP regimen (22.1%), and a combination of cyclophosphamide and vincristine (7.0%). In terms of monotherapy, the most common treatment regimens were etoposide (6%), mogamulizumab (4.9%), and methotrexate (3.6%). Mogamulizumab was used as first-line therapy by 8.3% of patients, either alone or with chemotherapy. These therapies were also the most common first regimens for each subcohort within the individual lines of treatment (**Table 2**).

**Table 2.** Treatment Regimens for Adult T-cell Leukemia/Lymphoma Patients in the First Line of Therapy

**Treatment Patterns**	**Overall Treated (N = 384)**	**1LOT (N = 144)**	**2LOT (N = 100)**	**≥3LOT (N = 140)**
Duration of continuous follow-up (months)				
Mean (SD)	15.0 (17.9)	10.4 (16.4)	14.8 (19.6)	19.9 (16.9)
Median [Q1, Q3]	8.2 [3.6, 19.2]	3.7 [2.0, 10.6]	6.6 [3.7, 15.3]	14.9 [8.8, 24.6]
First-line combination therapy, n (%)				
Cyclophosphamide, doxorubicin, vincristine (CHOP- and CHOP-like)	129 (33.6)	35 (24.3)	40 (40)	54 (38.5)
VCAP-AMP-VECP and VCAP-AMP-VECP like	85 (22.1)	18 (12.5)	26 (26.0)	41 (29.3)
Cyclophosphamide, vincristine	27 (7.0)	11 (7.6)	9 (9.0)	7 (5.0)
Cyclophosphamide, etoposide, vincristine	8 (2.1)	5 (3.5)	2 (2.0)	1 (0.7)
Cyclophosphamide, mogamulizumab, vincristine	6 (1.6)	3 (2.1)	1 (1.0)	2 (1.4)
Cyclophosphamide, vindesine	4 (1.0)	2 (1.4)	1 (1.0)	1 (0.7)
Etoposide, mogamulizumab	5 (1.3)	4 (2.8)	0 (0.0)	1 (0.7)
Cytarabine, methotrexate	4 (1.0)	4 (2.8)	0 (0.0)	0 (0.0)
Brentuximab vedotin, cyclophosphamide, doxorubicin	3 (0.8)	0 (0.0)	1 (1.0)	2 (1.4)
Carboplatin, cyclophosphamide, etoposide, mogamulizumab, ranimustine, vincristine, vindesine	2 (0.5)	1 (0.7)	0 (0.0)	1 (0.7)
Carboplatin, cyclophosphamide, etoposide, vincristine, vindesine	2 (0.5)	1 (0.7)	0 (0.0)	1 (0.7)
Carboplatin, doxorubicin, etoposide, ranimustine, vindesine	2 (0.5)	1 (0.7)	0 (0.0)	1 (0.7)
Cyclophosphamide, cytarabine, methotrexate, vincristine	2 (0.5)	0 (0.0)	1 (1.0)	1 (0.7)
Cyclophosphamide, doxorubicin	2 (0.5)	0 (0.0)	1 (1.0)	1 (0.7)
Cyclophosphamide, etoposide	2 (0.5)	2 (1.4)	0 (0.0)	0 (0.0)
Cytarabine, etoposide, methotrexate	2 (0.5)	1 (0.7)	0 (0.0)	1 (0.7)
First-line monotherapy, n (%)				
Etoposide	23 (6.0)	18 (12.5)	3 (3.0)	2 (1.4)
Mogamulizumab	19 (4.9)	10 (6.9)	3 (3.0)	6 (4.3)
Methotrexate	14 (3.6)	10 (6.9)	1 (1.0)	3 (2.1)
Cyclophosphamide	4 (1.0)	2 (1.4)	1 (1.0)	1 (0.7)
Lenalidomide	5 (1.3)	1 (0.7)	2 (2.0)	2 (1.4)
Carboplatin	3 (0.8)	2 (1.4)	0 (0.0)	1 (0.7)
Gemcitabine	2 (0.5)	2 (1.4)	0 (0.0)	0 (0.0)

Abbreviations: AMP, adriamycin (doxorubicin), manimustine (ranimustine), and prednisone; ATL, adult T-cell leukemia/lymphoma; CHOP, cyclophosphamide, doxorubicin, vincristine sulfate, prednisone; LOT, line of therapy; Q, quartile; SD, standard deviation; VCAP, vincristine, cyclophosphamide, doxorubicin, and prednisone; VECP, vindesine, etoposide, carboplatin, and prednisone.

Treatment regimens were defined as a segment of ATL-specific treatment that can be a monotherapy or combination therapy regimen (monotherapy: only 1 medication used without any other agents observed within a 30-day window; combination regimen: initiation of any other agent within 30-day window after the initial medication dispensing).

Line of therapy end date was defined as the earliest of the following events: discontinuation (eg, gap in treatment of ≥60 days), line advance (switch from index treatment to another ATL treatment within ≥30 days after line of therapy index treatment initiation) or augmentation (additional therapies initiated after the initial 30-day window within the line of therapy), end of continuous enrollment or end of study period.

Treatment regimens in the overall cohort were reported among patients with the respective number of treatment regimens observed (eg, overall cohort in 2LOT is among patients who had ≥2 lines of therapy).

In the second-line treatment (among patients who received ≥2 lines of therapy), the most common combination therapies utilized were CHOP and CHOP-like (8.6% of all patients), VCAP-AMP-VECP regimen (8.6%), and a combination of cytarabine and methotrexate (1.8%). For monotherapies in the second line, the most common regimens were mogamulizumab (8.9%), etoposide (7.0%), and lenalidomide (3.9%). Mogamulizumab-based therapy was the most frequently used second-line treatment, administered to total 55 patients (14.3%) **(Table 3)**.

**Table 3.** Treatment Regimens for Adult T-cell Leukemia/Lymphoma Patients in the Second Line of Therapy

**Treatment Patterns**	**Overall Treated (N = 384)**	**1LOT (N = 144)**	**2LOT (N = 100)**	**≥3LOT (N = 140)**
Second-line combination therapy, n (%)				
Cyclophosphamide, doxorubicin, vincristine (CHOP- and CHOP-like)	33 (8.6)	–	11(11.0)	22(15.8)
VCAP-AMP-VECP and VCAP-AMP-VECP like	33 (8.6)	–	11 (11.0)	22 (15.7)
Cytarabine, methotrexate	7 (1.8)	–	6 (6.0)	1 (0.7)
Etoposide, mogamulizumab	6 (1.6)	–	4 (4.0)	2 (1.4)
Cyclophosphamide, vincristine	5 (1.3)	–	2 (2.0)	3 (2.1)
Cytarabine, methotrexate, mogamulizumab	5 (1.3)	–	2 (2.0)	3 (2.1)
Cisplatin, gemcitabine	4 (1.0)	–	3 (3.0)	1 (0.7)
Cyclophosphamide, cytarabine, methotrexate, vincristine	4 (1.0)	–	0 (0.0)	4 (2.9)
Carboplatin, cytarabine, doxorubicin, etoposide, methotrexate, ranimustine, vindesine	3 (0.8)	–	2 (2.0)	1 (0.7)
Cyclophosphamide, etoposide, methotrexate	3 (0.8)	–	3 (3.0)	0 (0.0)
Brentuximab vedotin, cyclophosphamide, cytarabine, doxorubicin, methotrexate	2 (0.5)	–	1 (1.0)	1 (0.7)
Carboplatin, cytarabine, etoposide, methotrexate, vindesine	2 (0.5)	–	1 (1.0)	1 (0.7)
Carboplatin, etoposide, ifosfamide, mogamulizumab	2 (0.5)	–	1 (1.0)	1 (0.7)
Carboplatin, etoposide, mogamulizumab	2 (0.5)	–	0 (0.0)	2 (1.4)
Carboplatin, etoposide, mogamulizumab, vindesine	2 (0.5)	–	0 (0.0)	2 (1.4)
Carboplatin, gemcitabine	2 (0.5)	–	1 (1.0)	1 (0.7)
Cyclophosphamide, doxorubicin, vindesine	2 (0.5)	–	0 (0.0)	2 (1.4)
Cyclophosphamide, mogamulizumab, vincristine	2 (0.5)	–	1 (1.0)	1 (0.7)
Lenalidomide, mogamulizumab	2 (0.5)	–	1 (1.0)	1 (0.7)
Second-line monotherapy, n (%)				
Mogamulizumab	34 (8.9)	–	8 (8.0)	26 (18.6)
Etoposide	27 (7.0)	–	17 (17.0)	10 (7.1)
Lenalidomide	15 (3.9)	–	8 (8.0)	7 (5.0)
Methotrexate	11 (2.9)	–	2 (2.0)	9 (6.4)
Cytarabine	3 (0.8)	–	1 (1.0)	2 (1.4)
Tucidinostat	3 (0.8)	–	3 (3.0)	0 (0.0)
Gemcitabine	2 (0.5)	–	1 (1.0)	1 (0.7)

Abbreviations: AMP, adriamycin (doxorubicin), manimustine (ranimustine), and prednisone; ATL, adult T-cell leukemia/lymphoma; CHOP, cyclophosphamide, doxorubicin, vincristine sulfate, prednisone; LOT, line of therapy; Q, quartile; SD, standard deviation; VCAP, vincristine, cyclophosphamide, doxorubicin, and prednisone; VECP, vindesine, etoposide, carboplatin, and prednisone.

Treatment regimens were defined as a segment of ATL-specific treatment that can be a monotherapy or combination therapy regimen (monotherapy: only 1 medication used without any other agents observed within a 30-day window; combination regimen: initiation of any other agent within 30-day window after the initial medication dispensing).

Line of therapy end date was defined as the earliest of the following events: discontinuation (eg, gap in treatment of ≥60 days), line advance (switch from index treatment to another ATL treatment within ≥30 days after line of therapy index treatment initiation) or augmentation (additional therapies initiated after the initial 30-day window within the line of therapy), end of continuous enrollment or end of study period.

Treatment regimens in the overall cohort were reported among patients with the respective number of treatment regimens observed (eg, overall cohort in 2LOT is among patients who had ≥2 lines of therapy).

The most common monotherapies and combination therapies in the third line of treatment remained consistent with those seen in the second line, with CHOP and CHOP-like (4.4% of all patients), VCAP-AMP-VECP regimen (2.6%), and cytarabine and methotrexate (1.3%) being the most common combination therapies. In the third line, the most common monotherapy regimens were mogamulizumab (3.6%), etoposide (2.9%), and lenalidomide (2.9%). Mogamulizumab-based therapy was used as third-line therapy by 8.6% of patients **(Table 4)**.

**Table 4.** Treatment Regimens for Adult T-cell Leukemia/Lymphoma Patients in the Third Line of Therapy

**Treatment Patterns**	**Overall Treated (N = 384)**	**1LOT (N = 144)**	**2LOT (N = 100)**	**≥3LOT (N = 140)**
Third-line combination therapy, n (%)				
CHOP- and CHOP-like	17 (4.4)	–	–	17 (12.2)
VCAP-AMP-VECP and VCAP-AMP-VECP like	10 (2.6)	–	–	10 (7.1)
Cytarabine, methotrexate	5 (1.3)	–	–	5 (3.6)
Etoposide, mogamulizumab	4 (1.0)	–	–	4 (2.9)
Cisplatin, gemcitabine, mogamulizumab	3 (0.8)	–	–	3 (2.1)
Cyclophosphamide, mogamulizumab, vincristine	3 (0.8)	–	–	3 (2.1)
Cytarabine, methotrexate, mogamulizumab	3 (0.8)	–	–	3 (2.1)
Carboplatin, doxorubicin, etoposide, mogamulizumab, ranimustine, vindesine	2 (0.5)	–	–	2 (1.4)
Carboplatin, etoposide	2 (0.5)	–	–	2 (1.4)
Carboplatin, etoposide, ifosfamide	2 (0.5)	–	–	2 (1.4)
Cisplatin, gemcitabine	2 (0.5)	–	–	2 (1.4)
Cytarabine, lenalidomide, methotrexate	2 (0.5)	–	–	2 (1.4)
Gemcitabine, mogamulizumab	2 (0.5)	–	–	2 (1.4)
Lenalidomide, mogamulizumab	2 (0.5)	–	–	2 (1.4)
Third-line monotherapy, n (%)				
Mogamulizumab	14 (3.6)	–	–	14 (10.0)
Etoposide	11 (2.9)	–	–	11 (7.9)
Lenalidomide	11 (2.9)	–	–	11 (7.9)
Methotrexate	4 (1)	–	–	4 (2.9)
Tucidinostat	3 (0.8)	–	–	3 (2.1)

Abbreviations: AMP, adriamycin (doxorubicin), manimustine (ranimustine), and prednisone; ATL, adult T-cell leukemia/lymphoma; CHOP, cyclophosphamide, doxorubicin, vincristine sulfate, prednisone; LOT, line of therapy; Q, quartile; SD, standard deviation; VCAP, vincristine, cyclophosphamide, doxorubicin, and prednisone; VECP, vindesine, etoposide, carboplatin, and prednisone.

Treatment regimens were defined as a segment of ATL-specific treatment that can be a monotherapy or combination therapy regimen (monotherapy: only 1 medication used without any other agents observed within a 30-day window; combination regimen: initiation of any other agent within 30-day window after the initial medication dispensing).

Line of therapy end date was defined as the earliest of the following events: discontinuation (eg, gap in treatment of ≥60 days), line advance (switch from index treatment to another ATL treatment within ≥30 days after line of therapy index treatment initiation) or augmentation (additional therapies initiated after the initial 30-day window within the line of therapy), end of continuous enrollment or end of study period.

Treatment regimens in the overall cohort were reported among patients with the respective number of treatment regimens observed (eg, overall cohort in 2LOT is among patients who had ≥2 lines of therapy).

Nearly 74.7% of patients discontinued their initial line of treatment. Among them, 37.5% of patients discontinued from the 1LOT, 15.6% from the 2LOT, and 21.6% in the ≥3 LOT subcohort. According to the study definitions, patients in the 1LOT subcohort had only one treatment option available, so they could not switch between regimens. In contrast, 40% of patients in the 2LOT subcohort and 40.7% in the ≥3 LOT subcohort switched from their initial regimen to another ATL treatment. The median time from diagnosis to treatment initiation was 0.8 months. Only 13 patients (3.4%) in the treated cohort underwent stem cell transplant. Among these 13 patients, 6 (1.6%) were from ≥3 LOTs, 5 (1.3%) were from 2LOT, and only 2 (0.5%) were from 1LOT.

### Healthcare Resource Utilization and Treatment Costs

The treated cohort exhibited a high frequency of all-cause HRU in both medical and pharmacy services across all care settings, with nearly 96% of patients experiencing at least 1 hospitalization. Among those who were hospitalized, the average (SD) length of stay was 1.4 (1.3) months. Within the various LOT subcohorts, the percentage of patients with at least 1 hospitalization increased with the number of LOTs. The highest hospitalization rate was seen in the ≥3LOT subcohort where 98.6% of patients were hospitalized. This was followed by 2LOT subgroup at 96%, and 1LOT at 92.4%. ATL-related hospitalizations were reported in 93.2% of patients in the treated cohort, with a mean (SD) length of stay per hospitalization of 1.4 (1.3) months. Similar to the all-cause HRU findings, the proportion of patients with at least 1 hospitalization was higher in the 2LOT and ≥3LOT subcohorts than the 1LOT cohort.

Emergency room (ER) visits accounted for 29.9%, 27%, and 31.4% of patients who had at least 1 ER visit in the 1LOT, 2LOT, and ≥3 LOT subcohorts, respectively. Among 305 patients who had at least 1 visit to a physician’s office, the mean (SD) number of visits PPPM in the treated cohort was 1.8 (1.3), with no significant differences observed among the LOT subcohorts (1LOT, 1.9; 2LOT, 1.8; and ≥3 LOT: 1.7 visits). The proportion of patients with ATL-related ER visits was similar across all 3 LOT subcohorts.

The mean (SD) number of prescriptions PPPM in the treated cohort was 1.2 (1.2). Among the subcohorts, 1LOT subcohort had the highest number at 1.5 prescriptions, followed by 2LOT subcohort with 1.3, and ≥3LOT subcohort with 0.8, PPPM. Additionally, physician office visits and outpatient lab tests were notably high in both the treated cohorts and the subcohorts.

During the baseline period, the mean (SD) all-cause total healthcare cost PPPM for the treated cohort was $7010.9 ($53 940.6) [¥1 103 000 (¥8 486 600)]. The corresponding median cost was $1081.9 [¥170 200]. The mean (SD) all-cause total healthcare cost PPPM for the treated cohort (after initiation of ATL treatment) was $29 538.4 ($42 480.7) [¥4 647 300 (¥6 683 600)]. The corresponding median cost was $16 117.6 [¥2 545 300]. The overall mean (SD) cumulative healthcare cost per patient from diagnosis until the end of follow-up was $453 567.7 ($1 052 431.2) [¥71 359 800 (¥165 579 000)]. The corresponding median cost was $117 824.5 [¥18 537 300]. These costs were highest for the ≥3LOT subcohort ($708 635.4 [$1 274 132.4]; ¥111 489 600 [¥200 459 300]), followed by the 2LOT subcohort ($378 320.4 [$732 102.0]; ¥59 521 100 [¥115 181 600]), and 1LOT ($257 840.2 [$954 592.4]; ¥40 566 000 [¥150 186 000]) **(Table 5)**.

**Table 5.** All-Cause Healthcare Costs at Baseline and After Initiation of ATL Treatment in Overall Treated Cohort and Line of Therapy Subcohorts

	**Overall Treated (N = 384)**	**1LOT (N = 144)**	**2LOT (N = 100)**	**≥3LOT (N = 140)**
Baseline all-cause healthcare resource costs^a^ ($ PPPM)				
Mean (SD)	7010.9 (53 940.6)	3900.1 (10 068.3)	15175.0 (104 831.9)	4450.9 (9799.9)
Median [Q1, Q3]	1081.9 [429.9, 3263.9]	1086.4 [459.5, 3396.7]	754.7 [332.5, 2646.7]	1343.4 [481.6, 3470.4]
All-cause healthcare resource costs after initiation of ATL treatment ($ PPPM)				
Mean (SD)	29 538.4 (42 480.7)	21 970.2 (26 675.8)	29 903.4 (38 386.4)	37 062.0 (55 447.5)
Median [Q1, Q3]	16 177.6 [8783.9, 30 780.0]	14 048.3 [6656.6, 24 259.3]	18 215.3 [8330.7, 33 989.6]	18 546 [10 488.9, 35 862.7]
Cumulative^b^ all-cause healthcare resource costs per patient ($ PP)				
Mean (SD)	453 567.7 (1 052 431.2)	257 840.2 (954 592.4)	378 320.4 (732 102.0)	708 635.4 (1 274 132.4)
Median [Q1, Q3]	117 824.5 [52 332.1, 346 150.9]	62 740.9 [24 504.0, 117 411.0]	128 345.3 [53 165.4, 343 865.1]	245 615.4 [118 404.0, 685 689.8]
Baseline all-cause healthcare costs^a^ (¥1000 PPPM)				
Mean (SD)	1103.0 (8486.6)	613.6 (1584.1)	2387.5 (16 493.4)	700.3 (1541.8)
Median [Q1, Q3]	170.2 [67.6, 513.5]	170.9 [72.3, 534.4]	118.7 [52.3, 416.4]	211.4 [75.8, 546.0]
All-cause healthcare resource costs after initiation of ATL treatment (¥1000 PPPM)				
Mean (SD)	4647.3 (6683.6)	3456.6 (4196.9)	4704.8 (6039.4)	5831.0 (8723.6)
Median [Q1, Q3]	2545.3 [1382.0, 4842.7]	2210.2 [1047.3, 3816.8]	2865.8 [1310.7, 5347.6]	2917.9 [1650.2, 5642.3]
Cumulative^b^ all-cause healthcare resource costs per patient (¥1000 PP)				
Mean (SD)	71 359.8 (165 579.0)	40 566.0 (150 186.0)	59 521.1 (115 181.6)	111 489.6 (200 459.3)
Median [Q1, Q3]	18 537.3 [8233.4, 54 459.9]	9871.0 [3855.2, 184,72.3]	20 192.6 [8364.5, 54 100.3]	38 642.7 [18 628.5, 107 879.6]

Abbreviations: ATL, adult T-cell leukemia/lymphoma; LOT, line of therapy; PP, per patient; PPPM, per patient per month; Q, quartile; SD, standard deviation.

^a^Baseline costs: all-cause healthcare costs assessed from 6 months prior to ATL treatment initiation.

^b^Cumulative healthcare resource costs include all cause healthcare costs from ATL diagnosis to the end of follow-up.

The cost of outpatient visits and hospitalizations were the major cost drivers in all LOT subcohorts. Among the 3 LOT subcohorts, the mean (SD) cost of outpatient visits was highest in the ≥3LOT subcohort ($26 641.2 [$55 189.0]; ¥4 191 500 [¥8 683 000]), followed by the 2LOT subcohort ($18 056.2 [$36 235.4]; ¥2 840 800 [¥5 701 000]).

The mean (SD) total ATL-related healthcare cost PPPM for the overall cohort was $29 853.2 ($42 990.2) [¥4 696 800 (¥6 763 700)]. The corresponding median costs was $16 167 [¥2 543 600]. Mean (SD) total ATL-related healthcare costs were highest among the ≥3LOT subcohort ($37 241.4 [$56 201.0]; ¥5 859 200 [¥8 842 200]), followed by the 2LOT subcohort ($30 307.3 [$38 638.2]; ¥4 768 300 [SD, 6 079 000]). Among the HRU categories, outpatient visits ($20 050.6 [$43 519.6]; ¥3 154 600 [¥6 847 000]), hospitalizations ($14 637.9 [$17 933.2]; ¥2 303 000 [¥2 821 500]), and prescription drugs ($10 367.2 [$33 959.6]; ¥1 631 100 [¥5 342 900]) accounted for the most of the total ATL-related healthcare costs in the overall cohort. Similar to the all-cause healthcare costs, the mean (SD) costs for outpatient visits were highest in the ≥3LOT subcohort ($26 896.3 [$55 942.2]; ¥4 231 600 [¥8 801 500]), and the 2LOT subcohort ($18 412.6 [$36 625.9]; ¥2 896 900 [¥5 762 400]).

## DISCUSSION

To our knowledge, this is the first study that describes ATL treatment patterns based on lines of therapy and estimates HRU and associated costs in ATL patients using the MDV database. As it is one of the largest claims databases in Japan, the findings of this study provide deeper understanding of the real-world patient characteristics, treatment patterns, healthcare utilization, and costs of ATL patients in Japan. However, as with all retrospective claims-based studies, generalizability of the findings from this study should be interpreted with caution.

Among diagnosed ATL patients recorded as receiving systemic treatment during the follow-up period, 37.5% received only 1 line of therapy, 26% received 2 lines of therapy, and 36.5% were treated with 3 or more lines of therapy. The mean age of the overall cohort was approximately 65.4 years, while the mean age of the treated cohort was around 69.6 years. This is consistent with findings from a previously published real-world study involving ATL patients.^12^ In the 1980s, the reported male/female ratio of ATL patients was 1.4, indicating a male-predominant disease. However, this ratio declined to approximately 1 after the 1990s. Despite this, considering Japan’s general population male to female ratio of 0.95 in 2010, ATL is still regarded as a male-predominant disease in Japan.^6,12,25^ Interestingly, our study found that 55.2% of the patients in our overall cohort were female. This observation of female predominance may be attributable to the age structure and demographic composition of the MDV database, given that older populations generally include a higher proportion of women. Moreover, because claims-based data reflect HRU rather than true population incidence, selection bias may have contributed to the apparent female predominance. However, the available data does not permit determination of whether this finding reflects a genuine epidemiological trend or a database-related artifact. The most common comorbidities identified in our study included congestive heart failure, peptic ulcer disease, and mild liver disease. These conditions are frequently seen in the elderly Japanese population, and notably, 63.7% of the patient population in this study was 65 years or older.^26^

ATL subtype information was unavailable for 77.3% of cases, which is a key limitation of this study. Treatment for ATL varies by disease subtype, patient fitness status, treatment goals, and donor availability for allo-HSCT. In Japan, mostly “aggressive” subtype ATL patients receive aggressive chemotherapy regimens; a watchful waiting strategy is typically recommended for indolent, asymptomatic patients.^27^ However, disease progression can alter management in these patients. Nearly half of indolent cases may progress to aggressive disease over time; it is at that point when systemic treatment is typically initiated.^28^ In this study, of the ATL cases with known subtypes, 54% were indolent, which is higher than the proportion reported in the published literature (24.2%).^14^ A subgroup analysis was conducted to further evaluate the treatment differences among indolent, aggressive, and unknown ATL groups. Systemic therapy was administered to 15% of patients with indolent disease, 21% of those with unknown subtype, and 58% of those with aggressive disease. Among treated patients, the treatment regimens were largely consistent across subtypes. It is likely that the unknown subtype group predominantly consists of patients with indolent disease, explaining why low proportion of patients receive treatment in this group. However, this ascertainment cannot be validated without complete subtype identification due to database limitation. However, this limited treatment rate likely has minimal impact on regimen selection, as therapeutic approaches remained consistent among subtype groups.

For fit ATL patients with significant tumor burden, combination chemotherapy is considered the primary treatment aimed at curing the disease. There is no universally superior regimen for ATL, but the VCAP/AMP/VCEP protocols are commonly used in Japan, as supported by the JCOG9801 study. Although this study did not achieve its primary overall survival target, the VCAP/AMP/VCEP arm demonstrated better 3-year overall survival rates, improved progression-free survival, and higher complete response rates than the CHOP regimen. It is important to note that less than one-third of patients completed treatment in this study, and 3 treatment-related deaths were reported.^27,28^

In this study, CHOP or CHOP-like regimens were the most common first-line therapies for ATL, used in 33.6% of patients who received systemic chemotherapy. Following CHOP treatments, the VCAP-AMP-VECP regimen was used in 22.1% of patients as observed in the first-line treatment analysis. Patients in the ≥3LOT and 2LOT subcohorts were younger and likely were better able to tolerate aggressive therapies such as CHOP-like and VCAP-AMP-VECP-like regimens. In this study, 67.8% of ≥3LOT and 66.0% of 2LOT patients received these treatments as their first-line regimens, compared with 36.8% of patients in 1LOT subcohort. There was also a notable use of monotherapy among patients across all lines of treatment, with etoposide, mogamulizumab, methotrexate, cyclophosphamide, and lenalidomide being the most frequently utilized monotherapies. Mogamulizumab was administered as monotherapy or in combination therapy in 31.1% of patients across all treatment lines. These findings align with previously published literature from Japan.^29^

Only 3.7% patients received allo-HSCT in this study, which is low compared with published estimates from Japan (10%).^30^ It is likely that allo-HSCT is not sufficiently captured in the MDV database. In addition, the low number of allo-HSCT may reflect the limited pool of eligible candidates in the hospitals that contribute to the MDV database. According to the Japan Society for Hematopoietic Cell Transplantation, 929 allo-HSCTs were performed in ATL patients in Japan between 1995 and 2009, with a median patient age of 53 years.^31^ In the present study, the median age of ATL patients was 69 years, likely contributing to the low proportion of observed allo-HSCT. However, the reasons for this should be evaluated in future research in this patient population.

The median treatment durations for first-line, second-line, and third-line treatment regimens, including CHOP/CHOP-like and VCAP-AMP-VECP, were 3.7 months, 6.6 months, and 14.9 months, respectively, consistent with their established dosing schedules of 6 to 8 treatment cycles. Recently, valemetostat and tucidinostat were approved by the Japan Pharmaceutical and Medical Devices Agency for relapsed or refractory ATL.^32,33^ However, this study did not evaluate these newly approved therapies, as the study period ended in August 2022.

Discontinuation and switching of treatment regimens were common in the treated cohort, with the highest discontinuation rates occurring during the first line of therapy and the most switching seen in patients who had received 3 or more lines of therapy (≥3LOT subcohort). There are no published studies reporting discontinuation rates by line of therapy in patients with ATL, making comparisons challenging. Existing literature suggests that, in cancer patients treated with chemotherapy, treatment discontinuation and switching may be due to disease progression or adverse events.^34^ Reasons for discontinuation were not explored in this study. Some patients may have been classified as discontinued due to the end of continuous enrollment or the study period’s conclusion. The median follow-up for the 1LOT subcohort was only 3.7 months, which may not be enough time to evaluate treatment gaps in many patients. Additionally, stratifying discontinuation into more interpretable categories using claims-based proxies was not feasible in this study, making it difficult to interpret whether discontinuation represented poor tolerability, disease refractoriness, mortality, or artifacts of the algorithm to classify patients within various LOT subcohorts.

Information on HRU in ATL patients is limited due to its rarity. Also, studies reporting PPPM costs for Japanese ATL patients have not been previously published. The current study found that the treatment costs in ATL patients are significantly high in Japan, with the mean all-cause total healthcare costs PPPM being over 4 times greater ($29 538 vs $7011) than the corresponding baseline costs. Hospitalizations, outpatient visits, and prescription medications primarily contribute to these total costs. This is reflected in the cumulative cost disparities observed among the 3 LOT subcohorts. Patients within the ≥3LOT subcohort have a mean cumulative cost of $708 635.4, which is 2.7 times greater than the mean cumulative cost of $257 840.2 estimated for the 1LOT subcohort. This pronounced difference in cost is primarily attributable to increased hospitalizations, outpatient visits, and prescription medication requirements among patients in the ≥3LOT subcohort. In a real-world database study of peripheral T-cell lymphoma (PTCL) patients in the United States, most patients were hospitalized at least once during a median follow-up period of 2 years.^35^ In our study, 93.2% of ATL patients were hospitalized at least once during their follow-up for any ATL-related cause, with a mean length of stay of 1.4 months per inpatient admission.

Frequent outpatient visits are also common among ATL patients, both before and after intensive treatment, with current study indicating that these patients had an average of 1.8 visits per month. These outpatient visits significantly contribute to the total treatment cost burden, making up a significant proportion of the overall disease-related expenses. ATL patients receive various chemotherapy regimens, and the choice of subsequent lines of therapy substantially impacts the overall costs. Notably, we discovered that ATL-related HRU and costs represented 98.2% of the total all-cause healthcare costs, highlighting the considerable economic burden associated with an ATL diagnosis and its treatment. Within each LOT subcohort, ATL-related costs accounted for 98.0%, 99.3%, and 97.6% of the total all-cause costs in the 1LOT, 2LOT, and ≥3LOT subcohorts, respectively. A real-world study using the MDV database found that the all-cause total healthcare PPPM for PTCL was $20 282.^36^ In comparison, the healthcare cost for ATL was $29 538, which is 46% higher than the PTCL estimate. Although hospitalization, outpatient, and prescription costs were the primary cost components in both groups, outpatient visit costs and hospitalization costs were notably higher in ATL than in PTCL ($19 726 vs $12 600.9 and $14 513 vs $10 297, respectively).^36^ Finally, because costs were converted using a single exchange rate on 1 January 2025, some variation in the USD estimates may not fully reflect yen–US dollar fluctuations during the study period; however, this is unlikely to have materially affected the overall study findings.

As with all real-world studies, some limitations should be considered when interpreting the results of this research. This study faces inherent limitations due to its retrospective nature and reliance on a claims database, including potential misclassification of diagnoses, data entry and possible coding errors, and issues related to treatment discontinuation or switching. Patients were identified using ATL-specific disease codes, which may lead to some underestimation of cases. The requirement for at least 1 claim during the 6-month look-back period may have preferentially included patients with greater healthcare utilization, potentially introducing selection bias. Furthermore, MDV database records differ from traditional electronic healthcare records as they lack full connectivity across different providers. Each institution assigns a unique patient identifier, resulting in fragmented treatment histories when patients receive care from multiple facilities. This fragmentation complicates the creation of a comprehensive clinical picture and can result in duplicate records. Additionally, outpatient care from small clinics is often overlooked because these clinics typically do not submit data to the MDV database. Consequently, there is a lack of information for patients who receive care outside the participating network, including those who visit independent pharmacies, clinics, or hospitals that do not contribute data to the MDV.

Despite MDV being the largest claims database in Japan and the current study including 10 years of patient data, the rarity of ATL resulted in a small sample size of patients. The study cohort was further divided into subgroups based on the line of therapy, limiting the statistical power of the findings. Additionally, this study’s methodology contains several limitations inherent to the use of claims data. First, the algorithm used to define line of therapy relied strictly on treatment gaps, which may lead to misclassification; treatment interruptions due to side effects or patient health condition could also be misidentified as a transition to a new line of therapy. Reasons for treatment interruption or gaps in treatment cannot be ascertained when using the claims data; hence, it is difficult to interpret these treatment gaps precisely. Furthermore, the criteria for patient inclusion, requiring either 1 inpatient or 2 outpatient diagnoses (30 days apart), likely blended newly diagnosed individuals with those who had pre-existing disease (prevalent cases). While a 6-month washout period was utilized to identify newly treated patients, it is possible that these patients may have received systemic therapy prior to this window. This long washout period was utilized to minimize the likelihood of including prevalent cases. As treatment protocols for initial diagnoses often differ significantly from those used for relapsed or refractory ATL, this misclassification may have influenced the observed treatment patterns in the current study. An additional significant limitation of this study is its inability to evaluate clinical outcomes, such as treatment response or disease progression, owing to its observational design and reliance on claims-based data. Deaths are captured only when they occur during hospitalization. As a result, follow-up duration may be overestimated, and the treated cohort may be subject to survivorship bias. Patients censored at the end of continuous enrollment may have died outside the hospital, and such deaths may not be captured in this dataset.

## CONCLUSION

This study, utilizing the MDV database, provides a thorough real-world analysis of treatment patterns and associated HRU and costs for patients diagnosed with ATL in Japan. The findings confirm and quantify the significant clinical and economic burden of ATL, particularly among patients with aggressive subtypes or those who are progressing to later lines of therapy. High hospitalization rates, frequent outpatient utilization, and increasing cumulative healthcare costs across treatment lines underscore the need for more effective treatment strategies for ATL. Future studies investigating the impact of newly approved therapies may offer additional insights into the treatment patterns related to ATL. Given the rare nature of ATL, further research with more comprehensive patient data is necessary to fully capture the clinical outcomes associated with ATL diagnosis and treatment.

### Disclosures

Charles Dharmani, Zahid Islam, Pingping Qu, and Zoe Jiang are employees of Daiichi Sankyo, Inc. Lin Song is an employee of Daiichi Sankyo Co. Ltd. Jinjiang Wang, Li Li, and Yijing Tao are employees of DeltaMed Solutions, Inc. Charles Dharmani, Zahid Islam, Pingping Qu, and Zoe Jiang own restricted stock units of Daiichi Sankyo, Inc. The authors have no other relevant affiliations or financial involvement with any organization or entity with a financial interest in or financial conflict with the subject matter or materials discussed in the manuscript apart from those disclosed.
